# “Struggle to obtain redress”: Women’s experiences of living with symptoms attributed to dental restorative materials and/or electromagnetic fields

**DOI:** 10.3402/qhw.v11.32820

**Published:** 2016-12-09

**Authors:** Lena Mårell, Monica Lindgren, Kerstin Ternulf Nyhlin, Christina Ahlgren, Anders Berglund

**Affiliations:** 1 Department of Odontology, Faculty of Medicine, Umeå University, Umea, Sweden; 2 Västerbotten County Council, Umea, Sweden; 3 School of Life Sciences, University of Skövde, Skövde, Sweden; 4 Department of Community Medicine and Rehabilitation, Physiotherapy, Umeå University, Umea, Sweden

**Keywords:** Environmental intolerance, dental restorative materials, encounters, qualitative study

## Abstract

The aim of this study was to explore the experiences of illness and the encounters with health care professionals among women who attributed their symptoms and illness to either dental restorative materials and/or electromagnetic fields, despite the fact that research on health effects from dental fillings or electricity has failed to substantiate the reported symptoms. Thirteen women (aged 37–63 years) were invited to the study and a qualitative approach was chosen as the study design, and data were collected using semi-structured interviews. The analysis was conducted with a constant comparative method, according to Grounded Theory. The analysis of the results can be described with the core category, “Struggle to obtain redress,” the two categories, “Stricken with illness” and “A blot in the protocol,” and five subcategories. The core category represents the women's fight for approval and arose in the conflict between their experience of developing a severe illness and the doctors’ or dentists’ rejection of the symptoms as a disease, which made the women feel like malingerers. The informants experienced better support and confirmation from alternative medicine practitioners. However, sick-leave certificates from alternative medicine practitioners were not approved and this led to a continuous cycle of visits in the health care system. To avoid conflicting encounters, it is important for caregivers to listen to the patient's explanatory models and experience of illness, even if a medical answer cannot be given.

Environmental somatization syndrome, environmental illness, and environmental intolerance are various names of a syndrome first described during the 1940s. This syndrome is characterized by the patient's belief that his or her symptoms are caused by a very low exposure to chemical factors in the environment (Magill & Suruda, 1998). According to Göthe and Molin (Gothe, Molin, & Nilsson, 1995), patients with environmental intolerance and symptoms attributed to dental restorative materials and/or electromagnetic fields present multi-symptomatic conditions. Patients suffer from dizziness, fatigue, palpitations, headache, and musculoskeletal pain, as well as experience sleeping problems and concentration and memory disturbances (Tillberg et al., 2005). To date, research on the health effects of mercury released from dental amalgam and its subsequent uptake has failed to explain the clinical symptoms (Berglund, 1990; SCENIHR, 2008). Some studies of patients with environmental intolerance support the view of the syndrome as a psychosomatic disorder (Elander-Lindberg, Lindberg, & Larsson, 1994) or establish a vulnerable personality as the cause for the disorder (Bergdahl, Marell, Bergdahl, & Perris, 2005). Bergdahl and Bergdahl (2001) found that patients with environmental intolerance were more depressed, anxious, and stressed than healthy controls.

There is a problematic situation for patients living with an illness without a medical explanation. They have to work hard to find out various strategies to make the symptoms socially visible, real, and physical. Furthermore, they need to attempt to fit in with normative, biomedical expectations of correctness, especially when consulting doctors (Werner, Isaksen, & Malterud, 2004). In these consultations, where there is a strong contrast in perception of the disease between the patient and the doctor or dentist, patients often find the meetings disrespectful and perceive themselves dismissed by the doctors and will not accept a psychosomatic explanation of illness (Larun & Malterud, 2007). Furthermore, frustration is often evoked in the doctors or dentists when symptoms cannot be explained by traditional medical or dental training (Salmon, 2007). Such conflicting encounters have been described when biological markers are absent and a precise location of bodily symptoms cannot be described, which could lead to intensified symptoms and also prevent recovery (Larun & Malterud, 2007; Salmon, 2007). In such situations, the symptoms are often attributed to emotional factors or a failing personality (Malterud, 1987).

It is well known that explanations for medically unexplained or undefined disorders are more often given to women than men (Malterud, 1987, 1992). Women also represent about two-thirds of the patients who consult for oral and/or general symptoms attributed to dental restorative materials and/or electromagnetic fields (Langworth, Bjorkman, Elinder, Jarup, & Savlin, 2002). There is a need for a deeper understanding of women's experience of living with medically unexplained disorders, and to gain insight in their experience of the encounters with health care professionals. Therefore, the aim of this study was to explore the experiences of illness and encounters with health care professionals among a group of women with symptoms attributed to dental restorative materials and/or electromagnetic fields.

## Methods

A qualitative method with an emergent design was used as this is particularly well-suited for exploring phenomena that are poorly understood.

### Participants

Fourteen women who attributed their symptoms and illness to either dental restorative materials and/or electromagnetic fields were asked to participate in the study as informants. Twelve of them were referred to the Department of Oral Diagnosis, and two were asked to participate when they consulted their family doctor at a health care center in northern Sweden. One woman declined to participate before the interviewing started, so the study group consisted of 13 women. The criteria for participation were: (a) belief that symptoms were caused by dental restorations and/or electromagnetic fields and (b) no known signs of contact allergic reaction to dental materials. Furthermore, all women were thoroughly examined by their family doctor and dentists, without a medical explanation identified for their symptoms. The women had a mean age of 49 years (range 37–63 years). All of them worked in women-dominated occupations as teachers, nurses, or secretaries. At the time of the interviews, two of the women were on full-time sick leave and two were on part-time sick leave. The remaining women were working. Six women lived with a partner, two were single, and five were divorced. The reason for only interviewing women is that women represent about two-thirds of the patients referred for examinations of symptoms attributed to dental restorative materials and/or electromagnetic fields.

### Data collection

Semi-structured, individual interviews were used, according to Grounded Theory (Corbin, 1990; Glaser & Strauss, 1967), which meant that the collection of data with interviews and the analysis of data went on simultaneously. Four people interviewed the participants: a dentist, a senior lecturer in nursing, a general practitioner, and an occupational medicine physician. The interviews were informal and done with as few preconceptions as possible. On that occasion there were one interviewer and one respondent. During the interviews, the women were invited to talk freely and describe their situation in their own words. The interviews concentrated on themes about perceived symptoms, the woman's own explanation of her illness, treatments proposed by health care professionals, as well as the family and work situations. The themes were used as an interview guide by all four researchers. The respondents were interviewed once either at the health care center, the dental school, or at the participant's home, per the patients’ choice, and lasted between 1 and 4 h and were audio-taped and transcribed verbatim.

### Data analysis

The analyses were conducted according to Grounded Theory (Glaser & Strauss, 1967), that is, collection and analysis of data occurred simultaneously. All authors discussed the material with each other before the next interview. The transcript analyses were conducted in parallel with the interview process, in order to interpret data and saturate findings according to Grounded Theory (Glaser & Strauss, 1967). In Grounded Theory, there are two main characteristics in the methodology—the systematic and the constant comparative methods. The constant comparative method dictates that every part of the data, that is, emerging codes, categories, properties, and dimensions, is constantly compared with all others parts of the data in order to explore variations, similarities, and differences (Hallberg, 2006).

All four researchers carefully read each interview and discussed the emerging codes. After the reading and coding phase, constant comparisons were made, and the codes were categorized. Finally, we returned to the original data and re-read the parts that concerned symptom descriptions, illness experiences, and encounters with health care professionals.

In the beginning of the analysis, we experienced the interviews to be chaotic. We found the material complicated, and it was difficult to find coherence and to comprehend. It was also difficult to find the crux of the data. When the women talked freely, it was almost always only about their symptoms, and it was difficult to ask them about anything other than their illness. They described their symptoms over and over again. Their description of their symptoms was really convincing, and more severe than we ever expected.

### Ethical considerations

The participants received written and verbal information about the interviews, as outlined in the Helsinki Declaration (2008). In accordance with Swedish ethical law (SFS 2003:460), participation was voluntary and an informed consent was collected from all participants. The participants had the option to withdraw from the study at any time, which one woman did before the interviews started. The study was approved by the ethical committee in Umeå University, Umeå, Sweden.

## Findings

**Figure 1:**
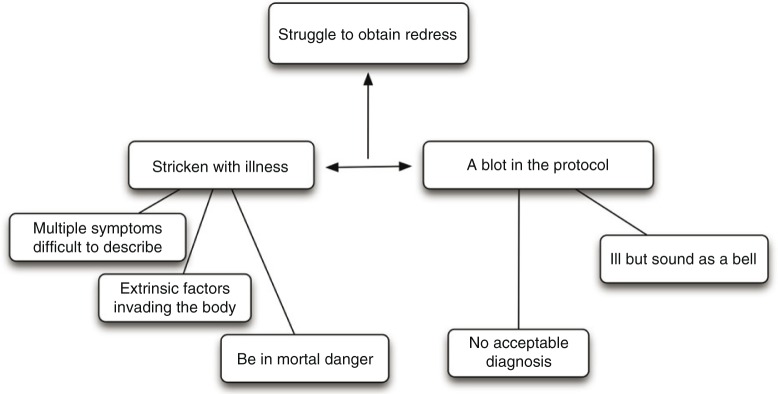
A model of the relation between the core category, the two categories, and the subcategories.

The analysis of the results of the interviews showed that most of the women had the impression that they had to “Struggle for redress,” and therefore, this became the core category. Other categories were also identified, “Stricken with illness” and “A blot in the protocol,” along with five subcategories. The relations between subcategories and the core category (Fig. 1) are further described and exemplified with quotes.

### Struggle to obtain redress

The core category, “Struggle to obtain redress,” represents the women's fight for resolution of the disagreement between their own experiences of a severe illness and rejection of a disease by doctors and dentists when all tests were normal. It describes the women's actions to avoid the stigma of being labeled hypochondriacs. The women had to struggle for referrals to specialists with the hope that further examinations would prove the women right. They described themselves as pioneers, standing on barricades, and were convinced that their symptoms would be described with a diagnosis that would give them approval. In this way, they hoped for redress.

### Stricken with illness

This category represents the women's experience of suffering from a life-threatening illness with multiple and diffused symptoms. The women were convinced that their symptoms were caused by external agents such as dental materials and/or electromagnetic fields. In most cases, they attributed the onset of their symptoms to a dental treatment. For that reason, all informants had their dental amalgam exchanged to other dental restorative materials and some of them even changed their dental materials twice. In spite of that, all of them still perceived illness; only three women experienced improvement of symptoms.

### Be in mortal danger

Intermittently, they had experienced powerful and severe physical symptoms that they felt they were going to die. The palpitations were so intense, the burning mouth so forceful, and the general weakness so strong, that they could not stand upright. However, they were happy to be alive, convinced of being afflicted by a deadly disease.

When she started her computer, my heart began to beat so fast that I felt I was going to die.

One of the women felt so ill after a dental treatment that she had to call for an ambulance.

### Multiple symptoms difficult to describe

All of the women gave powerful descriptions of a mixture of symptoms, which often were described in metaphors. They mentioned symptoms such as tiredness, sleeping disorders, infirmity, heart palpitations, digestion problems, spasmodic conditions, eye problems, tremor, vertigo, breathing problems, and difficulty concentrating, all in different combinations.

I got ache in the head, the neck and the back. My eyes turned red. I could hardly see. I got slime in my throat … and everything came at the same time.

I have a galvanic power in the jaw, it pulls in all directions.

Although, the women experienced intense symptoms, they found it difficult to describe them to the doctor. This made the symptoms difficult to understand, both for the women themselves and for their caregivers.

These vibrations went up and down in my body, it trembles inside the body.

### Extrinsic factors invading the body

The narratives focused on bodily symptoms, which were emphasized over and over again. They rejected psychological origins or life circumstances as causes for their symptoms as they experienced increased symptoms when visiting a dentist and/or being close to electric equipment such as computers and televisions.

When it got worse, I had a hard time at work. I also had an unusual situation at home, but that was still not a contributing factor. In fact, I was ill.

### A blot in the protocol

This category describes the women's experiences of encounters with doctors and dentists when they searched for help, treatment, and a reliable diagnosis. Although they felt severely ill, they perceived that they were being told they were physically healthy when no somatic pathology could be found. This made them feel as if the doctors and dentists saw them as hypochondriacs, and the women were scared of being classified as having a mental disorder.

### Ill but sound as a bell

Despite the experience of many troublesome symptoms, medical examinations and tests showed normal results, and the message from health care was that “if the test is normal, you should be healthy.”

I remember I was crying when I walked away from the doctor. I figured there was something wrong with me, but nothing was shown, all the investigations and tests showed nothing. They said that I'm healthy even though I feel like this!

When neither dentists nor doctors could find a medical diagnosis explaining the symptoms, it caused frustration and feelings of being “like a blot” in the medical records.

You only cause trouble. In fact, you are only a blot in the protocol.

### No acceptable diagnosis

The women continued to repeatedly consult new doctors and dentists in search of an explanation and a diagnosis that confirmed all their physical symptoms.

Without a physical diagnosis, the respondents became afraid that they would be treated as neurotics or malingerers if problems were discovered in their private lives.

It is nothing mental, you know. We know that we are right. That is the problem with us.

In contrast to medical and dental caregivers, alternative medicine immediately confirmed their illness with tests such as eye diagnosis. They gave quick and concrete answers and a somatic explanation. The women felt satisfied that someone had at last taken them seriously, even though they were not cured.

I got an appointment and he looked in my eyes and said ‘But woman, you are so poisoned. Your tongue and everything, I can't understand how you are sitting here’.

## Discussion

This study analyses women's experiences of living with a medically unexplained disorder attributed to dental restorative materials and/or electromagnetic fields.

The core category, “Struggle to obtain redress,” represents the informants’ fight for approval, when they experienced being rejected as malingerers by doctors and dentists although they were convinced that dental amalgam and electromagnetic fields made them sick. They saw themselves as pioneers, standing on a barricade and fighting for an explanation and a diagnosis of the illness, and that was the only way they could get redress.

Similar experiences of explanations of external factors causing illness being rejected by health care professionals have been described by women with musculoskeletal pain (Johansson, Hamberg, Westman, & Lindgren, 1999), chronic fatigue syndrome (Soderlund & Malterud, 2005), orofacial pain (Wolf, Birgerstam, Nilner, & Petersson, 2008), and fibromyalgia (Gustafsson, Ekholm, & Ohman, 2004). These patients experience themselves as victims of an unknown disease and consider themselves misjudged by doctors and dentists when they are not been given a physical explanation for their symptoms (Johansson et al., 1999; Soderlund & Malterud, 2005; Wolf et al., 2008) (Wolf, Birgerstam, Nilner, & Petersson, 2006). The same feelings of disgrace encountered by women in this study are summarized in the category “A blot in protocol” or like being a “spot” in the medical record.

It is possible that doctors and dentists interpreted the women's symptoms as panic disorder, since all tests were normal (Latas, Obradovic, & Pantic, 2009). But, being offered a psychiatric diagnosis was unacceptable for the informants. These divergent points of view on the symptoms’ origins constituted a basis for a conflicting encounter between the informants and the doctors and dentists.

**Figure 2:**
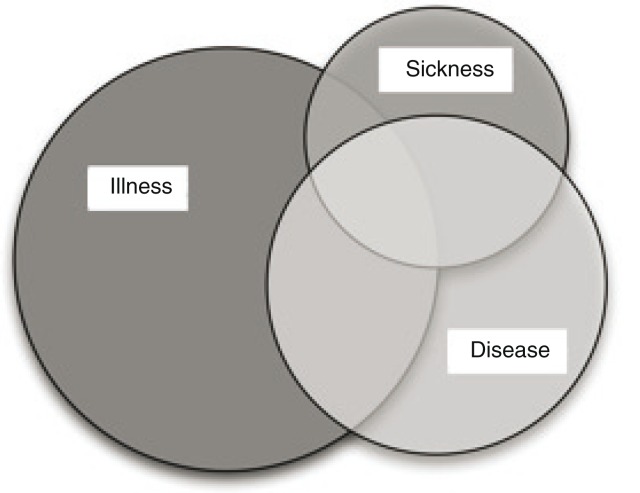
Relation between illness, disease, and sickness, modified from Wikman and Marklund.

A useful model to understand and describe the different views of symptoms by doctors, dentists, and patients could be the model by Wikman and Marklund (Wikman, Marklund, & Alexanderson, 2005). This model describes different aspects of ill health in terms of “illness,” “disease,” and “sickness.” The model (Fig. 2) describes illness as the self-identified ill health of a person based on self-reported symptoms. Disease is a condition verified with a diagnosis by medical experts and based on pathological findings. Sickness is related to the social role a person with disease or illness is given in society, for example, being given a sick-leave certificate (Wikman et al., 2005).

Thus, there could be varying degrees of overlap between the different areas. In patients with illness attributed to dental materials or electromagnetic fields, the overlap between the patient experience of illness and the doctors’ or dentists’ definition of disease seems to be small. In contrast, there are examples of diseases with a more defined diagnosis such as chronic heart failure with a high convergence between the patients’ experience of illness and the diagnostic criteria for disease. Also, type I Diabetes is a socially legitimized chronic disease with a biological explanation that is universally accepted (Thorne, Ternulf Nyhlin, & Paterson, 2000). These patients do not have to consult other health care providers to confirm a diagnosis and the symptoms will not be questioned.

The women in our study experienced more support and confirmation from caregivers in alternative medicine. Kristoffersen et al. (2016) concluded that alternative medicine was widely used in patients with health problems attributed to dental amalgam, as it is not an accepted diagnosis in the health care system. Clearly, alternative medicine has a broader and a more vague definition of disease; if you feel ill, choose to be ill, or have an illness behavior, then you have a disease (Johanisson, 1997).The definitions of illness and disease overlap to a greater extent in alternative medicine than in biomedical science, and this gives alternative medicine a great deal of authority to explain illness (Johanisson, 1997). Patients commonly combine alternative healing systems with biomedical care to provide alternative explanations of illness (Asbring & Narvanen, 2004). Kristoffersen et al. (2016) concluded that alternative medicine was widely used among patients with health problems attributed to dental amalgam.

However, explanations from alternative care were not accepted as a basis for a diagnosis or to provide a sick-leave certificate. Therefore, the informants had to consult medical care for new examinations, and this gives rise to a cycle in medical and dental care that may even exaggerate symptoms. A medical diagnosis from a doctor provides access to welfare benefits such as sick-leave certificates or disability pensions (Gustafsson et al., 2004). This makes it understandable why a diagnosis is extremely important for the patient (Johansson et al., 1999).

In an attempt to interrupt this pattern of numerous consultations, the model by Wikman and Marklund (Wikman et al., 2005) may be helpful, and can be used as a basis for discussions with patients. The patients can understand that their experiences form a different dimension of the model than the one which allows diagnoses, and that their experiences are neither questioned nor are they blamed for the knowledge gap. This could create trust in the encounter and offer a base for recovery. Definitions of illness, disease, and sickness, and what they represent, are not immutable, but they change over time (Johanisson, 1997). Sjursen et al. (2015) suggested that the most important aspects of the amalgam controversy were found in the difference between the rational understanding of multifactorial explanations of health and the emotions around the questions about dental amalgam. This underscores the importance of health care professionals learning more about how patients think, act, and feel regarding illness attributed to dental restorations.

In Sweden, reports of health problems attributed to dental amalgam have decreased from the 1980s, and it is now a relatively small problem. But still, the fear of mercury poisoning has caused some patients to replace their amalgam fillings despite research on toxic effects of mercury released from amalgam having failed to establish a causal relationship between the mercury exposure and the symptoms presented by these patients (Melchart et al., 2008; SCENIHR, 2008). Furthermore, the effect of these replacements has been insufficiently evaluated, and it has been shown that amalgam removal is not the only treatment option for improvement of health (Melchart et al., 2008). However, studies on self-rated health show that replacement of dental restorative materials has the largest impact on alleviation of symptoms, which is somewhat contradictory (Tillberg, Marell, Berglund, & Eriksson, 2008). The patients experienced an improvement in their over-all health despite the results showing that the number of symptoms had increased after removal of dental restorations. Both psychosocial and environmental conditions as well as lifestyle factors seem to be related to poor self-rated health (Molarius et al., 2007). In Sweden, the use of mercury-containing products, among others, in dental amalgam, has been prohibited since July 2008 for environmental reasons. But dental amalgam will still be present in people's mouths for the next 20–30 years. Since many dental restorative materials contain substances that may be perceived as toxic and in the future there might be dental materials other than dental amalgam that are suspected to cause side effects.

Symptoms attributed to dental restorative materials and/or electromagnetic fields seem to be multifactorial with dental, medical, social, and psychological factors involved. Therefore, doctors and dentists must cooperate when examining and treating patients with these symptoms.

### Methodological considerations

The aim of this study was to explore experiences of illness and encounters with health care professionals. Therefore, a emergent research design was chosen which explores structures, mechanisms, and social phenomena that affect human beings. The abstract knowledge obtained by this type of research may be generalized to other social contexts that share similar structures (Gustafsson et al., 2004). Furthermore, qualitative research strategies make it possible to explore social events as experienced by individuals in their natural context (Johansson, Risberg, & Hamberg, 2003). Established concepts such as validity, reliability, objectivity, and generalization cannot be used in qualitative research. Alternative criteria for scientific rigor, initially introduced by Lincoln and Guba, are presented—credibility, dependability, confirmability, and transferability (Hamberg, Johansson, Lindgren, & Westman, 1994).

To increase the credibility of this study, we used researchers from different disciplines for both data collection and analysis. Researchers with different preconceptions and understandings created high-quality discussions in the analysis phase. A potential disadvantage and limitation is that the interviews were not conducted by one person, but by four different people, probably resulting in some differences in the way interviews were conducted. However, the interviews were done with an emergent design and the analyses went on simultaneously, and all four researchers read and coded all of the interviews.

## Conclusion

In consultations, caregivers feel frustrated when they can neither understand nor help patients with environmental intolerance and symptoms attributed to dental materials. Based on the informants’ descriptions, conflicts could have arisen from the fact that doctors or dentists did not understand the patient's experience of illness. Therefore, it is important to listen to the patient's explanatory model and try to understand that they have a strong feeling of illness. Caregivers must attempt to provide a better consultation and treatment, as this could help avoid an escalation of symptoms. Patients with environmental intolerance seek an explanation of their illness. Even if a medical answer cannot be given, an illness story and a positive consultation can be created, which could contribute to recognition and provide a sense of coherence for the patients.
